# Inconsistent response of taxonomic groups to space and environment in mediterranean and tropical pond metacommunities

**DOI:** 10.1002/ecy.3835

**Published:** 2022-10-05

**Authors:** Ángel Gálvez, Pedro R. Peres‐Neto, Andreu Castillo‐Escrivà, Fabián Bonilla, Antonio Camacho, Eduardo M. García‐Roger, Sanda Iepure, Javier Miralles‐Lorenzo, Juan S. Monrós, Carla Olmo, Antonio Picazo, Carmen Rojo, Juan Rueda, María Sahuquillo, Mahmood Sasa, Mati Segura, Xavier Armengol, Francesc Mesquita‐Joanes

**Affiliations:** ^1^ Cavanilles Institute of Biodiversity and Evolutionary Biology, University of València Paterna Spain; ^2^ Department of Biology Concordia University Montreal Quebec Canada; ^3^ Instituto Clodomiro Picado, Facultad de Microbiología Universidad de Costa Rica San José Costa Rica; ^4^ Emil Racovitza Institute of Speleology Cluj Napoca Romania; ^5^ Subdirecció General del Medi Natural Generalitat Valenciana València Spain; ^6^ Museo de Zoología, Centro de Investigación en Biodiversidad y Ecología Tropical Universidad de Costa Rica San Pedro Costa Rica

**Keywords:** dispersal limitation, environmental selection, freshwater metacommunity, multitaxon analysis, tropical and temperate ecology

## Abstract

The metacommunity concept provides a theoretical framework that aims at explaining organism distributions by a combination of environmental filtering, dispersal, and drift. However, few works have attempted a multitaxon approach and even fewer have compared two distant biogeographical regions using the same methodology. We tested the expectation that temperate (mediterranean‐climate) pond metacommunities would be more influenced by environmental and spatial processes than tropical ones, because of stronger environmental gradients and a greater isolation of waterbodies. However, the pattern should be different among groups of organisms depending on their dispersal abilities. We surveyed 30 tropical and 32 mediterranean temporary ponds from Costa Rica and Spain, respectively, and obtained data on 49 environmental variables. We characterized the biological communities of bacteria and archaea (from the water column and the sediments), phytoplankton, zooplankton, benthic invertebrates, amphibians and birds, and estimated the relative role of space and environment on metacommunity organization for each group and region, by means of variation partitioning using generalized additive models. Purely environmental effects were important in both tropical and mediterranean ponds, but stronger in the latter, probably due to their larger limnological heterogeneity. Spatially correlated environment and pure spatial effects were greater in the tropics, related to higher climatic heterogeneity and dispersal processes (e.g., restriction, surplus) acting at different scales. The variability between taxonomic groups in the contribution of spatial and environmental factors to metacommunity variation was very wide, but higher in active, compared with passive, dispersers. Higher environmental effects were observed in mediterranean passive dispersers, and higher spatial effects in tropical passive dispersers. The unexplained variation was larger in the tropical setting, suggesting a higher role for stochastic processes, unmeasured environmental factors, or biotic interactions in the tropics, although this difference affected some actively dispersing groups (insects and birds) more than passive dispersers. These results, despite our limitations in comparing only two regions, provide support, for a wide variety of aquatic organisms, for the classic view of stronger abiotic niche constraints in temperate areas compared with the tropics. The heterogeneous response of taxonomic groups between regions also points to a stronger influence of regional context than organism adaptations on metacommunity organization.

*Tropical species often exhibit patchy geographic distributions that respond to no obvious relations with climate or habitat*

MacArthur, 1972

## INTRODUCTION

Ecological communities are not isolated systems, as they were often considered in the past, but are instead components of large spatial networks connected by dispersal, known as metacommunities (Hanski & Gilpin, 1991; Wilson, 1992). The development of the metacommunity theory was fostered by the establishment of four main archetypes (i.e., species sorting, mass effects, patch dynamics, neutral theory) by Leibold et al. (2004), based on previous ecological theories. This allowed new research avenues, soon testing whether empirical data fitted these archetypes. These tests mostly showed a higher enforcement of niche‐related versus spatial effects (these attributed to dispersal limitation), although the latter could also be important, especially at large spatial scales (Cottenie, 2005; Soininen et al., 2007). The use of the paradigmatic archetypes, however, has been criticized on the basis of their restricted, exclusive views as extreme patterns, so that ecologists should better focus on the underlying mechanisms structuring metacommunities (Winegardner et al., 2012). These mechanisms can be classified into four broad sets, involving selection, dispersal, speciation and drift (Leibold & Chase, 2018; Vellend, 2010). Recently, Thompson et al. (2020) slightly modified Vellend's (2010) list of main processes; in addition to ignoring speciation for tractability, these authors suggested to split selection‐based processes in two types: those depending on density, related to biotic interactions, and those being density independent, affected by abiotic conditions. In addition, these authors suggested to apply the stochasticity involved in ecological drift to all density independent or dependent processes, such as dispersal, therefore embedding demographic changes affected by different mechanisms.

Research in the past two decades has established that the processes driving metacommunity organization depend on complex interactions between local patch attributes (and their landscape settings) and species attributes. In this framework, higher connectivity among patches may increase dispersal, homogenizing the metacommunity, and allowing habitat matching for low dispersers, whereas environmental heterogeneity among sites may be essential for species selection and increased beta diversity (Castillo‐Escrivà, Aguilar‐Alberola, & Mesquita‐Joanes, 2017; Erös et al., 2017; Grönroos et al., 2013). Focusing on the organisms traits, it has been suggested that body size, dispersal mode, or trophic level may influence the outcome of metacommunity organization, so that large active dispersers or those with small propagules may be less affected by spatial barriers than passive dispersers or than those with larger propagules (Astorga et al., 2012; De Bie et al., 2012; Vanschoenwinkel et al., 2010), although these relationships can be strongly dependent on the environmental context (Soininen, 2014).

To detect, differentiate and understand the roles of different processes on (meta)community assembly has been particularly challenging because of the complex ways that landscape and species characteristics may affect local patches and dispersal between them. Despite the complexity in which assembly processes may proceed, these are often conceptualized as taking place continuously on different spatial scales (Peres‐Neto et al., 2012; Viana & Chase, 2019): large‐scale processes (including biogeography; Leibold et al., 2010) that regulate the movement of organisms among local communities (e.g., landscape heterogeneity, connectivity, dispersal limitation); and fine‐scale processes that regulate the success of species following either their own arrival or the arrival of other species (e.g., niche differentiation, local environment, microhabitat heterogeneity). Consequently, our understanding of metacommunities is, more often than not, scale dependent (e.g., stream, basin, and ecoregion; Heino et al., 2015; Leibold & Chase, 2018). For instance, small spatial scales, with dispersal surplus and/or homogeneous environmental conditions, favor metacommunity homogeneity, whereas at large spatial scales, dispersal rates decrease, generating metacommunity variation consistent with dispersal limitation and wider environmental gradients. Notwithstanding the potential increase in environmental gradients with spatial extent, both small and large spatial scales may however reduce the strength of environmental selection and/or environmental tracking, which should be more relevant at intermediate scales. This is because intermediate scales may be large enough to encompass heterogeneous environments, yet allowing moderate dispersal rates to track suitable localities (Heino et al., 2015). As such, keeping spatial extent constant is somewhat essential for contrasting metacommunity structure across different landscapes and/or taxonomic groups (as spatial effects also depend on the dispersal abilities of each organism; Wiens, 1989). Few studies, however, have explored metacommunity structure contrasting distinct biogeographical regions (with large differences in their abiotic settings and biota) while controlling for spatial extent (Myers et al., 2013), or have used a multitude of taxonomic groups (e.g., Beisner et al., 2006; Heino et al., 2017). None of them however, as far as we know, combined in the same empirical test a large taxonomic gradient with distant biogeographical regions to check if context dependency effects may overlay taxon‐specific responses.

The expectation of more uniform environmental conditions in tropical regions (at least through time) should lead to reduced species sorting compared with the more heterogeneous environment in temperate areas (Leibold & Chase, 2018). Indeed, some studies have found that temperate communities were more environmentally controlled than tropical ones for a given group of organisms (Myers et al., 2013; Souffreau et al., 2015). For aquatic habitats, massive floods during the rainy season increase aquatic connectivity in tropical areas (Bunn et al., 2006; Junk et al., 1989; Larsen et al., 2019), reducing dispersal limitation and homogenizing local communities (Brasil et al., 2020; Thomaz et al., 2007), in contrast with the more intense isolation of water bodies in the mediterranean‐climate regions. In both tropical and mediterranean systems, we may then expect that metacommunity structure is a result of dynamic switches between dispersal limitation and environmental filtering through time (Fernandes et al., 2014; Jacobson & Peres‐Neto, 2010), which could exert stronger effects on aquatic ecosystems of dry temperate areas than in the wetter tropical regions.

Metacommunity studies focusing on distinct organisms within the same landscape and localities have revealed strong differences among taxa regarding spatial versus environmental effects (Beisner et al., 2006; Brasil et al., 2020; Gálvez et al., 2020; Padial et al., 2014), largely interpreted as affected by mechanisms of dispersal limitation versus selection, respectively, and defined by Vellend (2010). Variation in dispersal mode and body size are important features associated with dispersal capacity and, consequently, modulating the strength of environmental filtering and dispersal limitation (De Bie et al., 2012). For active dispersers, dispersal ability increases with body size, making it easier for large organisms to spread among suitable sites (favoring species sorting), therefore avoiding dispersal limitation (Csercsa et al., 2019; Grönroos et al., 2013). Passive transport, conversely, may limit the dispersal capacity of large‐sized organisms in contrast with small ones (which also produce more propagules; Finlay, 2002). As a result, environmental selection should, in principle, be stronger for small organisms (Van der Gucht et al., 2007) although the evidence about this association is mixed (e.g., Heino et al., 2012; Schulz et al., 2012; Soininen, 2014). Despite the large differences between several published studies, it seems that environmental selection is a proportionally stronger process than dispersal for the organization of freshwater metacommunities, especially in smaller organisms. However, some groups such as diatoms, ostracods, or macroinvertebrates show high variability in the role of these processes (Gálvez et al., 2022). Altogether, dispersal capacity, species traits, geographic variation in environmental features, and biogeographic context should interact in structuring metacommunities (please refer to Leibold et al., 2010 for a discussion).

One way to address the complexity in which local communities assemble and metacommunity dynamics proceed is by determining whether: (1) different taxa (including completely different life cycle strategies, e.g., bacteria vs. birds) assemble differently within the same landscape; or (2) different environmental settings (landscapes) structure different taxa in similar ways. To answer these questions, one needs to study the metacommunity structure of multiple taxa across different landscapes. Although this approach can provide strong inferences about the generality (or lack thereof) of assembly mechanisms and whether contingencies are due to landscapes or species attribute differences, there are obvious logistic challenges. In this study, we set out an ambitious empirical study to contrast the relative importance of environmental versus spatial factors on structuring metacommunities. We sampled temporary pond metacommunities in an extremely wide range of taxonomic groups (across 24 groups of organisms in total, varying from bacteria to vertebrates) across two very distinct biogeographic regions (Neotropical and Mediterranean) within the same temporal span and encompassing the same spatial extent within regions, and based on the same sampling design, protocols, and data measurements. We tried to encompass as much information on environmental variability as possible, including data from both aquatic and surrounding terrestrial habitats (Likens & Bormann, 1974; Sardans et al., 2012). For that purpose, we carried out an intense effort on (mainly abiotic) environmental characterization, including macroclimate, landscape, and local waterbody variables. In both study regions, Costa Rica and Spain, precipitations are distributed seasonally, with a marked dry season that promotes the formation of temporary ponds. These are valuable ecosystems for metacommunity studies due to their well delimited spatial and temporal boundaries, and their isolation within a terrestrial matrix (at least for aquatic organisms). We framed our study around three predictions: (1) both environmental and spatial effects should have lower influences on metacommunity structure of tropical in contrast with mediterranean ponds; (2) different taxa should respond differently within regions but similarly between regions; and in particular (3) metacommunity structure should be related to the variation of dispersal abilities among taxa, in which higher environmental filtering should occur for small passive dispersers and large‐bodied active dispersers in contrast with smaller active dispersers.

## METHODS

### Study area

**FIGURE 1:**
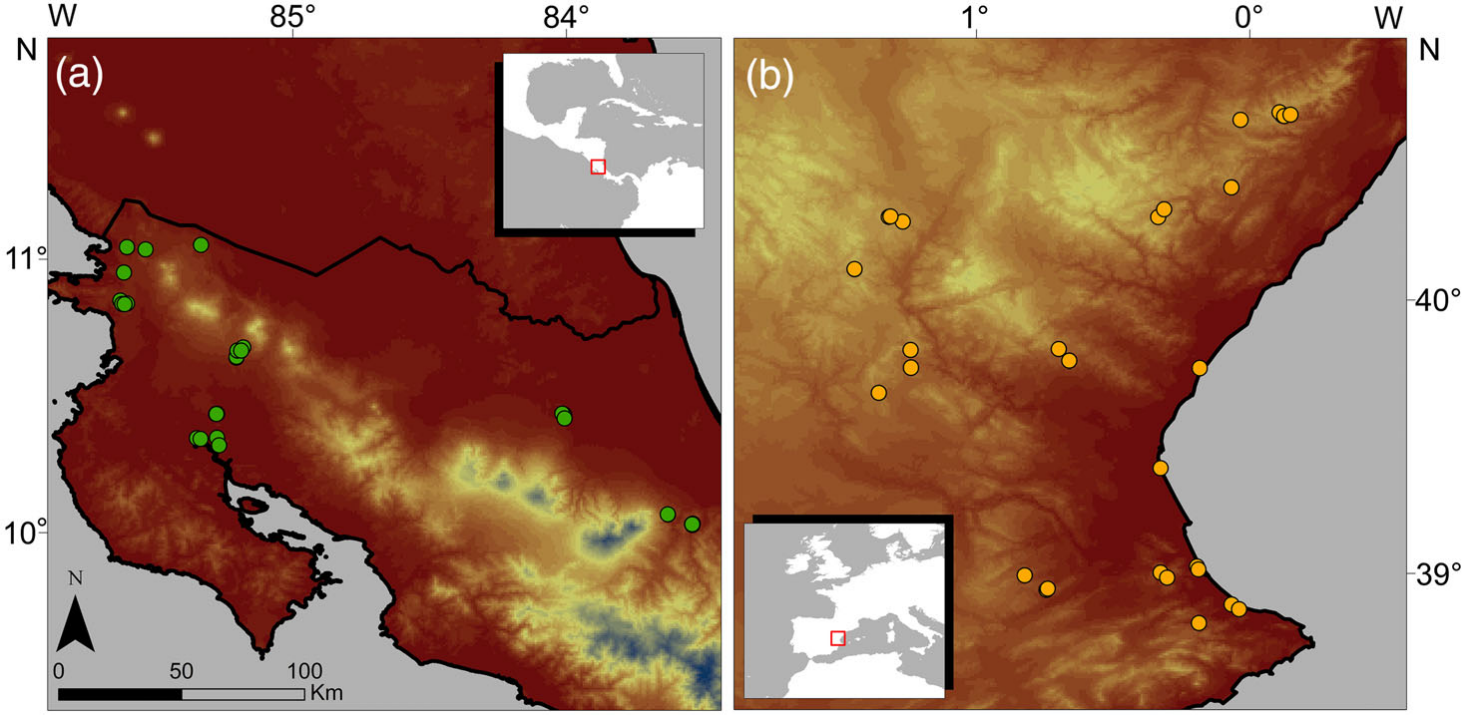
Map of the study area with the location of the ponds sampled in each region: Costa Rica (a) and eastern Spain (b).

We looked for well preserved temporary freshwater bodies from sea level up to 1500 m above sea level (asl) within a similar spatial extent in two distant biogeographic regions (Neotropical and Mediterranean) with dry tropical and mediterranean climates respectively. We selected 30 tropical and 32 mediterranean (seasonal or semipermanent) temporary ponds in a study area of 13,000 km^2^ in eastern Spain and 10,000 km^2^ in northern Costa Rica. The maximum distances between ponds were 209 and 252 km in Spain and Costa Rica, respectively (Figure 1). The selected ponds encompass different typologies including peripheral areas of coastal wetlands, inland shallow lakes, interdunal slacks, and small naturalized farmland ponds. All ponds were shallow (<2 m), fresh to oligohaline waters (<7000 μS/cm) and varied in their hydroperiod lengths, from 6 months to some years. The tropical ponds experienced almost constant warm temperatures during the whole year (24.9 ± 1.3°C) and high but variable precipitation (2486 ± 934.7 mm). The mediterranean ponds were in a region with warm annual mean temperature (12.9 ± 3.0°C) and reduced annual precipitation (537 ± 68.3 mm) (Fick & Hijmans, 2017). We sampled the ponds at the beginning of the hydroperiod (2 weeks after infilling, during the rainy season), in May 2017 for the selected tropical ponds and January 2018 for the mediterranean ponds.

### Environmental characterization

We measured limnological variables in situ including water transparency, which was measured with a Snell tube (Van de Meutter et al., 2006), as well as water temperature, pH, electrical conductivity, and dissolved oxygen concentration, determined using a WTW Multi 3430 Multiparameter Meter (with WTW SenTix 940 for pH, TetraCon 925 for conductivity and FDO 925 for oxygen concentration). Unfiltered water samples were taken for further volumetric analyses of chloride and alkalinity, and filtered water for photometric determination of ammonium, nitrite, nitrate, sulfate and phosphate (SpectroquantMerk and AquaMerk test kits and T90 + UV/VIS Spectrophotometer) in the laboratory. The water used for nutrient content analyses was filtered in situ through Whatmann GF/F filters. We extracted chlorophyll *a* from these GF/F filters with acetone 90% and analyzed the extract using spectrophotometry (Jeffrey & Humphrey, 1975). Hydrogeomorphological variables included maximum and average depth, measured with a graduated stick, maximum and minimum diameter, surface area, and shoreline development, as DL = *L*/(2 $\sqrt{\pi A}$), where *L* = perimeter and *A* = area (Aronow, 1982), using Google Earth Pro 7.3.2.5776 (Google Inc.). We took six random sediment samples at different depths to analyze the granulometry (following De Vaasma, 2010), and organic and carbonate content (according to Heiri et al., 2001); we estimated the proportion of dry weight represented by different grain sizes (>2, 2–1, 1–0.5, 0.5–0.25, 0.25–0.063, 0.063–0.036, <0.036 mm), and by organic matter and mineral carbonates. We estimated the hydrological regime (seasonal or semipermanent) and the main water origin (rain, streams or phreatic inputs) using information from local experts, direct observation and maps. Biotic variables (in addition to chlorophyll *a*) included the percentage of surface area of the pond covered by submerged, floating and emergent vegetation (visually estimated), the presence of fish, and the presence of livestock. Landscape metrics were estimated using Google Earth (resolution ~1 m^2^), including elevation asl and the percentage of different types of land cover (agricultural fields, low grass, high grass, scrub, forest, or buildings) in a circular buffer of 100 m in diameter around the sampling point (Gálvez et al., 2020; Pedersen et al., 2006). Land cover diversity was measured using the Shannon index of the different land cover types. Finally, macroclimatic variables were extracted from WorldClim (resolution 30 arcseconds; Fick & Hijmans, 2017) to obtain mean annual, maximum and minimum temperatures, temperature range, mean annual precipitation, and precipitation seasonality for each pond, using ArcGIS 10.3 software (ESRI, 2014).

### Biological communities

For each pond, we characterized prokaryotic (Bacteria and Archaea), phytoplankton, zooplankton, benthic invertebrate, amphibian, and bird communities. For prokaryotic organisms, two samples were obtained: a 1 L sterilized bottle with pond water and a microtube with 2 ml of pond wet sediment. Afterward, in the laboratory, we filtered the water through 3‐μm pore polycarbonate filters (Nucleopore, Whatman) to remove larger particles. The remaining water was filtered again through 0.2 μm pore polycarbonate filters (Nucleopore, Whatman) to concentrate samples for subsequent DNA analysis. Filters and sediment samples were cold stored in microtubes filled with RNAlater reagent, until further processing. DNA extractions, PCR, and bioinformatics for taxonomic assignments were made following Picazo et al. (2019).

Phytoplankton samples were obtained directly from the water column at the center of the pond (100 ml stored in amber‐colored glass bottles) and fixed with 3 ml of Lugol's solution. Phytoplankton taxa were identified to species level whenever possible following mainly Huber‐Pestalozzi (1976–1982) and Wolowski and Hindák (2005). Zooplankton samples were taken dragging a hand net (63‐μm mesh size) through 10–20 m of water column, whenever possible, integrating all different microhabitats and depths. Samples were fixed in 4% formaldehyde (final concentration, v/v) and identified at the species level, whenever possible, following Koste (1978) and Segers (1995) for rotifers, Alonso (1996), Elías‐Gutiérrez et al. (2008), and references therein for branchiopods, Dussart (1967, 1969) and Elías‐Gutiérrez et al. (2008) for copepods, and Błędzki and Rybak (2016) for both branchiopods and copepods.

Benthic invertebrate communities were sampled using a hand net (20 × 20 cm^2^, 250‐μm mesh size). In total, ~10 m (if ponds were large enough) were sampled across all microhabitats. Samples were fixed in 96% ethanol and invertebrates identified to the lowest taxonomic rank possible, mostly following Wiederholm (1983), Tachet et al. (2010), Thorp and Covich (2010), and Springer et al. (2010), and references therein, for macroinvertebrates, and Meisch (2000), Karanovic (2012), and references therein for Ostracoda.

The occurrence of amphibian species in the ponds was registered in situ by noting the presence of eggs, tadpoles, adults, and calls. Due to the low species richness and the easy detectability of species in the mediterranean ponds, we surveyed each pond with a hand net (800 cm^2^, 2‐mm mesh pore) with a constant effort of 10 min, and we examined the surroundings of the pond for an additional 10 min (Wilkinson, 2015). When in situ identification of larvae was not possible, we identified larvae in the laboratory by their oral disk. In tropical ponds, with higher species richness and lower detectability, we performed night surveys looking for individuals in the surroundings of each pond with an effective effort of up to 2 h per pond (the sum of the total effort by all the surveyors; Heyer et al., 1994), avoiding full moon nights. Adult specimens were identified visually or by calls. Bird surveys were performed using single 15‐min point counts per pond with one surveyor, in which we recorded species presence identified either visually (up to a maximum distance of ~100 m) or by their calls (Ralph et al., 1995).

### Statistical analyses

We quantified the role of environmental and spatial effects, by partitioning metacommunity variation (Peres‐Neto et al., 2006) of each taxon and region between environmental variables and spatial functions. The groups considered were archaea from the sediment, archaea from the water, bacteria from the sediment, bacteria from the water, phytoplankton, Rotifera, microcrustaceans, macroinvertebrates, Amphibia, and Aves. We also analyzed subgroups of these organisms separately due to major taxonomic and trophic differences among them. Phytoplankton was therefore split as Cyanobacteria, Chlorophyceae, Bacillariophyceae (diatoms) and mixotrophic flagellate phytoplankton (including Chrysophyceae, Cryptophyta, Euglenophyta and Dinoflagellata). Microcrustaceans were split into Branchiopoda, Copepoda and Ostracoda, and macroinvertebrates into Mollusca and Insecta. Moreover, insects were divided into Palaeoptera (including Odonata and Ephemeroptera), Heteroptera, Coleoptera and Diptera (considering also Chironomidae separately as another subgroup). Separately, groups and subgroups accounted for 24 species matrices based on presence–absence data for each region.

For each group (and subgroup) and region, we performed variation partitioning via generalized additive models (GAMs; Wood, 2011), recently used to explore metacommunity variation (Viana et al., 2021). Despite its popularity in community analyses, the performance of redundancy analyses (RDA) is weak when species respond nonlinearly to environmental variation (e.g., Makarenkov & Legendre, 2002). To tackle its limitations, we used GAMs as a nonlinear modeling framework considering both environmental and spatial variables, and allowing a better fit to the data.

Response matrices in GAMs were composed of predicted values of the presence–absence data, obtained from generalized linear latent variable models (GLLVM with binomial distribution) with the lowest Akaike information criterion (AIC; Niku et al., 2017). This allowed us to concentrate on the common sources of variation among species and reduce computational time from extracting GAMs for a large number of taxa. Prior to these analyses, environmental variables were inspected and transformed either logarithmically or with the arcsine of the square root, depending on the distribution of the raw data, to reduce skewness and leverage of extreme values (McDonald, 2014). When applying GAMs, due to the small sampling size (30 tropical and 32 mediterranean ponds), we were limited to a maximum number of three variables, to allow GAMs generate nine splines per variable (3 variables × 9 splines = 27), maximizing the response of the selected variables. As a consequence, before executing GAMs (using a quasibinomial distribution), we reduced the number of environmental predictors using the first three principal components (PCs) in a principal components analysis (PCA) of all variables. These three PCs accounted for 47.17% and 49.19% of the measured environmental variation of the mediterranean and tropical ponds respectively. As spatial predictors, we used spatial functions (the default splines in the *mgcv* R package) calculated from longitude and latitude coordinates.

We reduced the number of environmental and spatial predictors (three PCs, longitude and latitude) introduced in GAMs by means of forward selection, with a double‐stopping criterion (Blanchet et al., 2008). First, we selected only significant variables (*p*‐value < 0.05). Second, the adjusted *R* ^2^ of the selected variables had to be lower than the adjusted *R* ^2^ of the model with the whole set of variables. This procedure was repeated for the environmental variables (three PCs) and spatial variables (latitude and longitude) separately. We included at least one environmental or spatial variable in the rare cases that the double‐stopping criterion was not satisfied, as long as the selected variable was significant. If more than three variables were selected, we manually reduced the number of variables (either spatial or environmental) by removing the selected variables that less contributed to explaining the observed variation, until reaching the maximum number of three selected variables. As a result, GAMs provide information on the total proportion of metacommunity variation explained by environmental (E) and spatial (S) factors together (E + S) and separately, estimating the pure environmental fraction (E|S; often associated with environmental selection), the pure spatial fraction (S|E; that most authors interpret as variation due to dispersal, but please refer to Gilbert & Bennett, 2010; Smith & Lundholm, 2010; Livingston et al., 2017), and the overlap of environment and space (E∩S; which represents environmental variation that is spatially structured). The unexplained proportion or residuals represent common and uncommon sources of variation not explained by (measured) environmental and spatial variation, usually interpreted as sampling issues, stochasticity driving to ecological drift, unmeasured biotic or abiotic factors, or interactions among factors (Leibold & Chase, 2018).

The contribution (*R* ^2^) of environmental variation to species distributions (i.e., species sorting via environmental selection) can be inflated when residuals and response variables (i.e., species distributions here) are both autocorrelated. Traditionally, ecologists remove the spatialized component of the environment via variation partitioning, focusing then solely on the E|S fraction. This procedure, however, may reduce estimates of the importance of environmental selection, because even the spatialized component of the environment that may not bias estimates of variation partitioning is eliminated from the E|S fraction (please refer to Clappe et al., 2018 for a discussion). To assess the importance of environmental drivers (spatialized and nonspatialized), we used the correction method for the environmental component as described in Clappe et al. (2018). This method produces unbiased estimates of the environmental contribution even under spatial autocorrelation of residuals. To make results comparable among taxa and region, we transformed the fractions of explained variation into the relative proportion of purely environmental effects (E|S/(E + S)), purely spatial effects (S|E/(E + S)) and a spatially correlated environment (E∩S/(E + S)). Finally, we carried out a test of homogeneity of multivariate dispersion (PERMDISP; Anderson, 2006) to contrast the environmental heterogeneity of both regions (tropical vs. mediterranean) using the whole set of measured environmental variables, as well as a matrix of just limnological variables (which are expected to be more local) and a matrix of just macroclimatic variables (which are expected to vary at regional scale, and to be more spatially correlated). All analyses were performed in R (v4.0.2; R Core Team, 2021) and using R packages *vegan* (Oksanen et al., 2019), *ade4* (Bougeard & Dray, 2018), *adespatial* (Dray et al., 2021), *gllvm* (Niku et al., 2020), and *mgcv* (Wood, 2017). Environmental characterization and community matrices can be found in Figshare (doi.org/10.6084/m9.figshare.14644608.v4). Codes for variation partitioning analyses can be found at this link: doi.org/10.5281/zenodo.6883482. More details about the environmental framework of each set of ponds are provided in Olmo et al. (2021).

## RESULTS

**FIGURE 2:**
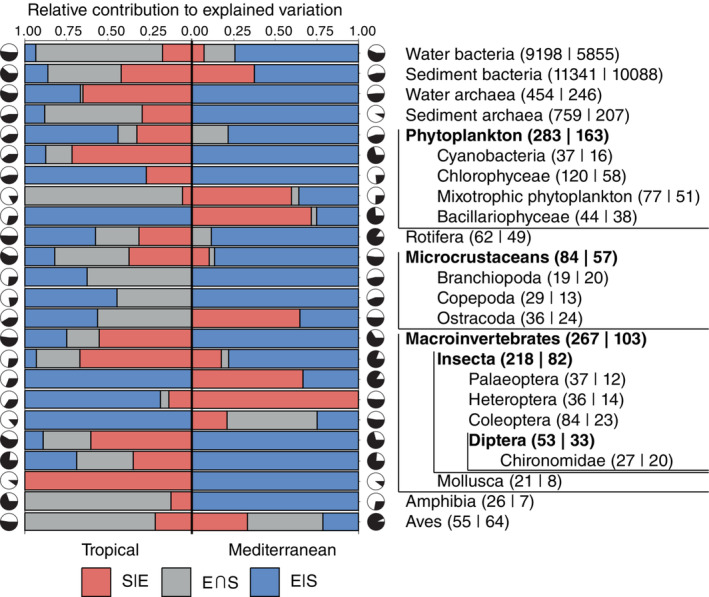
Results of variation partitioning analysis for each group of organisms in tropical and mediterranean metacommunities. The relative contribution of pure environment (E|S/(E + S)), spatialized environment (E∩S/(E + S)) and space (S|E/(E + S)) are represented by a different color. Black portions in pie charts represent the total proportions of explained variation, and white portions represent the residual variation. Groups in bold type include species from the groups enclosed in the corresponding following indented line(s). Number of identified taxonomic units, that is, species in most groups, are shown between parentheses (tropical|mediterranean) next to each group label.

**FIGURE 3:**
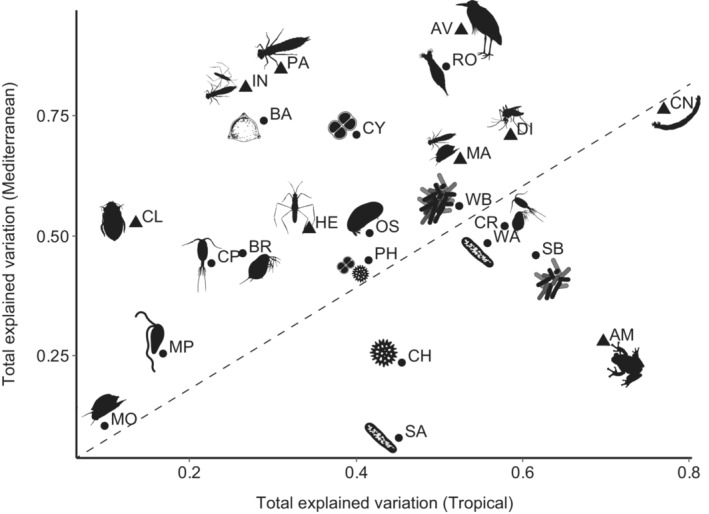
Relationship between the total explained variation (E + S) in the tropical and the mediterranean metacommunities. Dashed line represents the 1:1 theoretical correspondence of the total explained variation in both regions. Passive dispersers are represented by circles, whereas active dispersers are represented by triangles. AM, Amphibia; AV, Aves; BA, Bacillariophyceae; BR, Branchiopoda; CH, Chlorophyceae; CL, Coleoptera; CN, Chironomidae; CP, Copepoda; CR, microcrustaceans; CY, cyanobacteria; DI, Diptera; HE, Heteroptera; IN, Insecta; MA, macroinvertebrates; MO, Mollusca; MP, mixotrophic flagellate phytoplankton; OS, Ostracoda; PA, Palaeoptera; PH, phytoplankton; RO, Rotifera; SA, sediment archaea; SB, sediment bacteria; WA, water archaea; WB, water bacteria. Silhouettes obtained from PhyloPic.org.

**FIGURE 4:**
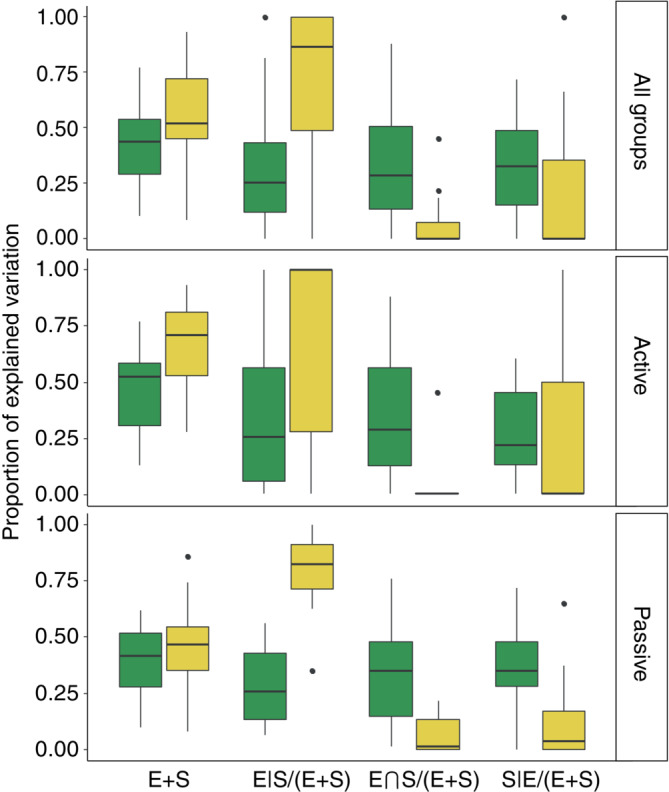
Total explained variation (E + S) for all groups or separately for passively and actively dispersing organisms. Relative contribution to total explained variation of the pure environmental component (E|S/(E + S)), the spatialized environmental component (E∩S/(E + S)), and the pure spatial component (S|E/(E + S)), omitting those groups with E + S in the fourth quartile. Green and yellow colors represent tropical and mediterranean ponds, respectively.

The total proportion of explained variation by environmental and spatial components (E + S) ranged from 0.10 for mollusks to 0.77 for chironomids in the tropical ponds (average 0.42 ± 0.18). In the mediterranean ponds, the explained variation varied from 0.08 for sediment archaea to 0.93 for birds (average 0.54 ± 0.23). Results of these variation partitioning analyses are shown graphically in Figure 2 and are detailed in Appendix S1: Table S1 and Figure S1. The E + S proportion for each taxonomic group was usually very different when comparing tropical with mediterranean ponds (Figure 3). Only a few groups, including water archaea and bacteria, phytoplankton, cyanobacteria, mixotrophic phytoplankton, microcrustaceans, and ostracods had similar proportions of explained variance for both regions, but there were a larger number of groups, particularly insects and birds, with much higher explained variance in mediterranean than in tropical ponds. Only prokaryotes from the sediment, chlorophycean algae and amphibians had a markedly higher E + S proportion in the tropical ponds. Overall, E + S proportions were higher in the mediterranean metacommunities compared with the tropical ones, but this difference was mostly due to differences between some groups of active dispersers (insects and birds), whereas passively dispersing metacommunities seem to have a similar E + S proportion both in tropical and mediterranean ponds (Figure 4). The only group of active dispersers that had lower E + S in the mediterranean ponds were the amphibians (Figure 3).

**FIGURE 5:**
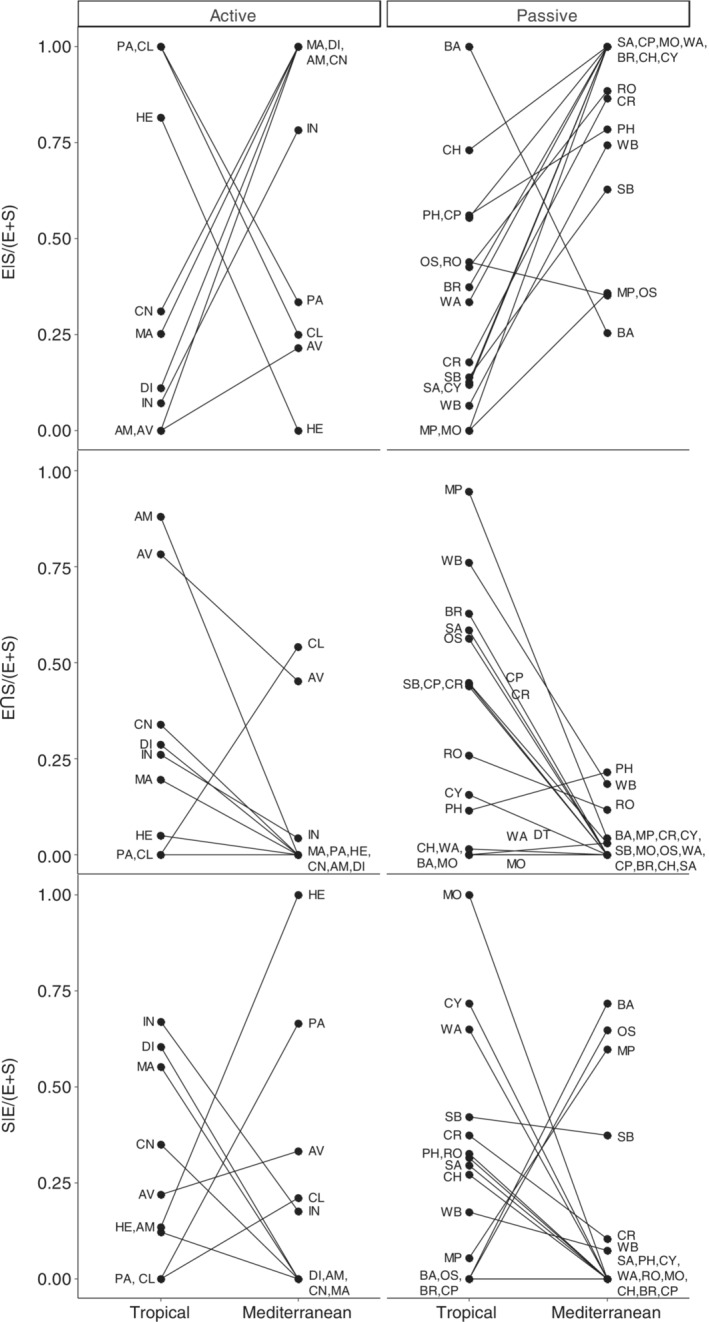
Relative contributions to total explained variation by the pure environmental component (E|S/(E + S), spatialized environment (E∩S/(E + S)) and pure spatial effects (S|E/(E + S)), for each group of organisms, and according to geographic region and dispersal mode. Black lines connect the same groups from different regions. Group codes as in Figure 3.

The groups with very low E + S values in one region usually showed a relationship to only one pure fraction (either E|S or S|E) (Figure 2) in that region, which accounted for 100% of the total explained variance (please refer to, e.g., mollusks in both regions or beetles in the tropical region; Figure 2). This might be related to the analytical methods used, so that no more summarizing variables would be selected at low values of explained variance. Therefore, to avoid biases for further graphical explorations of the data regarding E|S/(E + S) or S|E/(E + S), we excluded them from some calculations (Figure 4). We found overall higher pure environmental effects (E|S) in mediterranean compared with in tropical metacommunities (Figures 2, 3, 4, 5). In contrast, the pure spatial fraction (S|E) was generally higher in the tropical metacommunities, despite some mediterranean groups exhibiting an important spatial control (Ostracoda, Heteroptera, Palaeoptera, diatoms and the mixotrophic flagellate phytoplankton), and surprisingly some of these (Heteroptera, Palaeoptera) were apparently more influenced by environment in the tropical setting (Figure 5). The response of most groups was very different when comparing tropical and mediterranean settings; only ostracods showed a similar E|S fraction between both regions, and bacteria and birds for S|E (in addition to a few groups with values of zero for these fractions) (Figure 5). The overlap of environment and space (E∩S) was greater in tropical ponds, and usually very low in the mediterranean metacommunities (Figure 4). The total environmental fraction (E) was higher in the mediterranean‐climate region, whereas total spatial fraction (S) was higher in the tropical region (Figure 4). Pure environment (E|S) explained a higher proportion of variance than purely spatial component (S|E) in mediterranean ponds, whereas pure spatial and pure environmental effects were similar in tropical ponds.

Pairwise comparisons for each taxonomic group between regions showed that the pure environmental fraction generally played a greater role in mediterranean groups than in their tropical counterparts (Figure 5), and this pattern was especially strong for passive dispersers, but not so clear for active ones. Pure spatial effects were important in structuring metacommunities of even small organisms such as algae, bacteria, or archaea. Regarding the spatially correlated environment and the pure spatial fraction, we found tropical groups to be more influenced by these effects compared to their mediterranean homologous groups, with most exceptions to this pattern occurring in active dispersers (Figure 5), such as insects and birds. The relative contributions of these components did not follow any clear trend on body (or propagule) size, neither for active nor passive dispersers. When we compared the relative contributions of space and environment between merged groups of passive and active dispersers (Figure 4), we found similar fractions explained by purely environmental, spatially correlated environmental and spatial effects in passive and active tropical dispersers, although the latter showed higher variability. Despite large differences in environmental and spatial contributions in the mediterranean ponds, both passive and active dispersers behaved similarly, although mediterranean passive dispersers were more affected by pure environmental factors, and more distant in that matter from the tropical groups, than active ones. Environmental effects were therefore generally higher in mediterranean than in tropical ponds, whereas the spatial component was higher in the tropical than in the mediterranean‐climate region. This pattern was most clearly seen in passive rather than in active dispersers (Figure 4), whose wider variability in explained variance for the different components makes the comparison less straightforward.

Results of PERMDISP showed nonsignificant differences in environmental heterogeneity between regions for the whole environmental dataset (*p*‐value = 0.303; 6.159 average distance to centroid in mediterranean ponds, 5.613 in tropical ponds). However, mediterranean ponds showed significantly higher limnological heterogeneity (*p*‐value = 0.002; 3.113 average distance to centroid in mediterranean ponds, 1.752 in tropical ponds), whereas tropical ponds exhibited significantly higher climatic heterogeneity (*p*‐value = 0.005; 1.311 average distance to centroid in tropical ponds, 0.921 in mediterranean ponds).

## DISCUSSION

In this study, we set out to contrast the environmental and spatial contributions on metacommunity structure among a wide range of taxa inhabiting temporary ponds in two distant biogeographic regions, Neotropical and Mediterranean, with large differences in climate and biodiversity. Overall, we found a lower proportion of explained variation in tropical than in mediterranean pond metacommunities, considering both environmental and spatial effects across the diverse array of studied organisms. Notwithstanding the limitation of our study comparing only two areas, these results are consistent with the expectation that more productive environments in the tropics, with less variable and higher temperatures, might be more stochastic in community assembly than less productive ones (Gomes‐Mello et al., 2021), with higher temperature fluctuations, such as ponds in mediterranean areas, where metacommunities might be modulated more tightly by environmental determinism (Chase, 2010). The greater explanation of metacommunity variance in mediterranean ponds seem to be mostly related to active dispersers (e.g., insects, birds), with the exception of amphibians. Possibly, larger demographic stochasticity (not linked to environmental changes), together with the higher diversity of the tropical fauna (and therefore higher number of uncommon species), hampered the match of environmental and/or spatial patterns to species distributions in these habitats (Leibold & Chase, 2018).

Our results showed that both environmental and spatial effects, usually interpreted mainly as environmental selection and dispersal processes respectively (but please refer to Gilbert & Bennett, 2010; Livingston et al., 2017; Smith & Lundholm, 2010), played an important role in the assembly of pond metacommunities in both regions for most groups. However, pure environmental effects were stronger in mediterranean compared with tropical pond metacommunities, as previously found for vegetation and bacterioplankton metacommunities (Myers et al., 2013; Souffreau et al., 2015). Given the higher limnological heterogeneity across the studied mediterranean water bodies, these results were therefore not unexpected (Ai et al., 2013), as larger gradients can generate more environmental space for divergently specialized organisms. So, even if there were more species in the tropical ponds, given their shorter environmental gradient and reduced species sorting, we would expected to find a higher degree of ecological redundancy and stochasticity in these ponds. However, we cannot discard unmeasured important environmental factors or intense biotic interactions, which could play important roles in aquatic metacommunities (García‐Girón et al., 2020) and can be particularly strong in tropical environments (Leibold & Chase, 2018; Roslin et al., 2017).

We hypothesized that spatial effects would be lower in the tropical metacommunity, due to a shift in connectivity during the rainy season, which should lead to community homogenization (Brasil et al., 2020; Rojo et al., 2016; Thomaz et al., 2007), in comparison with the reduced precipitation in the mediterranean region, which is expected to make ponds more isolated from one another. Unexpectedly, we found the opposite pattern. Low spatial effects in mediterranean ponds might reflect historical connectivity due to transhumance (Incagnone et al., 2014) or intense movements of potential vectors including birds and mammals among ponds (Frisch et al., 2007; Valls et al., 2016, 2017; Vanschoenwinkel et al., 2008). The strong spatial effects in the tropical metacommunity might also be partly explained by the sampling period, given that, at early stages of the hydroperiod, connectivity was not yet at maximum. It is possible that during the early stages of the hydroperiod, dispersal constraints might be equally strong for aquatic organisms in both regions. Additionally, annual floods during the wet season drive connectivity changes, homogenizing both environmental conditions and biological communities (Brasil et al., 2020; Thomaz et al., 2007). Therefore, early hydroperiod spatial patterns in small tropical organisms can be the result of historical contingencies (Castillo‐Escrivà, Valls, et al., 2017b), such as sequential flooding events (or heavy rain periods) that shifted connectivity at the regional scale. These events may increase the spatial structure of passively dispersed organisms in more connected areas compared with other distant and more isolated regions.

In addition to hydrology, orography could be a stronger dispersal barrier in the tropical than in the mediterranean metacommunity. Dispersal of tropical species, adapted to homogeneous temperature regimes, could be more limited by sharp altitudinal climate shifts than temperate species, adapted to a wider range of temperatures (Janzen, 1967). The tropical ponds in our study are distributed across two watersheds separated by the Continental Divide, with wide differences in rainfall amount and seasonality, which can have varying impacts on the distributions of some species (Chandler & King, 2011). This orographic barrier and the associated abrupt climatic transition are probably characterized by the shared components between environmental and spatial components (E∩S), which were found to be higher for the tropical ponds, where climatic heterogeneity was also higher. Therefore, reduced limnological gradients and orographic barriers can lead to dispersal limitation (Janzen, 1967) at larger scales, and higher regional connectivity can lead to dispersal surpluses (Brasil et al., 2020; Heino et al., 2015; Thomaz et al., 2007) at smaller scales in the tropical area. These two processes could explain the proportionally greater role of space in tropical rather than in mediterranean metacommunities.

Some studies have found that the degree of variation of spatial versus environmental effects on metacommunity structure strongly depends on propagule size and the dispersal mode of organisms (Beisner et al., 2006; De Bie et al., 2012; Heino et al., 2015). However, we found it to be very variable for the same group between geographical settings (i.e., mediterranean vs. tropical). A significant spatial effect was found even in the smallest studied organisms, including prokaryotes, planktonic protists, and small metazoans. Organisms smaller than 1 mm have traditionally been considered ubiquitous, lacking biogeographical effects (Finlay, 2002), although more recent analyses suggested that there was no strong support for or against this statement (Fenchel et al., 2019). In this sense, we found significant spatial effects in both active and small passive dispersers, including bacteria and archaea, supporting the view that microorganisms are subjected to basically the same processes as those affecting macroorganisms (Hortal, 2011). For instance, the effects of extreme isolation on dispersal limitation allow for allopatric differentiation even in bacterial subspecies, as has been shown for Maritime Antarctic lakes (Hahn et al., 2015). By contrast, some groups that should exhibit strong spatial control, such as coleopterans or mollusks (e.g., De Bie et al., 2012), were apparently mostly controlled by the environment in some of our studied metacommunities (but their total explained variance was frequently very low). Indeed, we did not find the expected differences in spatial and environmental effects between different body‐sized, active or passive dispersers from the same region (De Bie et al., 2012; Padial et al., 2014), as neither did Heino et al. (2012) nor Schulz et al. (2012). Only a few groups have shown similar proportions of purely spatial and (purely plus spatially correlated) environmental effects in both regions, including bacteria from the water column and the sediment, branchiopods and copepods. Both bacteria from the water column and the sediment, which are supposed to have higher dispersal ability than branchiopods and copepods, showed higher spatial effects in both metacommunities. Considering only pure environmental factors, there is only one group, the Ostracoda, that had a similar response between regions. These dissimilar results across regions, also differed from those found by other multitaxon studies (Beisner et al., 2006; De Bie et al., 2012; Gálvez et al., 2020; Padial et al., 2014), suggesting the impossibility to extract general patterns on environmental and spatial influence on metacommunity structure according to organism dispersal ability. Nevertheless, the results obtained for the mediterranean metacommunity are somehow in tune with those found in the abovementioned similar studies (e.g., stronger role of purely environmental effects in bacteria, phytoplankton, rotifers, microcrustaceans or macroinvertebrates than purely spatial effects), among others (Gálvez et al., 2022). By contrast, these patterns were much more diffuse in the tropical metacommunity, or even inverted.

Passive and active dispersers within the same region barely differed between them in their spatial and environmental effects, suggesting that spatial and environmental constraints are similar for organisms with different dispersal abilities (Schulz et al., 2012), and rather depend strongly on context. Large fractions of community variation, however, remain unexplained. This suggests an important role for the elements of chance or randomness (Jeffries, 1989), but also unmeasured (biotic or abiotic) environmental effects, or interactions between effects (Leibold & Chase, 2018). On the one hand, stochastic demographic processes and priority effects are expected to be strong at the beginning of the hydroperiod due to egg‐bank hatching, before abiotic and biotic sorting of species can effectively act as filters in structuring local communities (Antón‐Pardo et al., 2016; Castillo‐Escrivà, Valls, et al., 2017c; Mahaut et al., 2018). This may result in increased unexplained variation, with model residuals accounting for a higher proportion in the tropical metacommunity, probably related to its higher species richness, but also because of lower limnological heterogeneity (Leibold & Chase, 2018), as shown by the homogeneity test of multivariate dispersion. Higher limnological heterogeneity may therefore account for a stronger influence of environmental selection processes in the mediterranean ponds, whereas in the richer tropical communities, under shorter environmental gradients, we may expect a higher influence of ecological redundancy. On the other hand, biotic interactions are known to be important mechanisms structuring metacommunities (García‐Girón et al., 2020), especially in the tropics where these interactions are presumably stronger than at higher latitudes (Roslin et al., 2017; Zvereva & Kozlov, 2021). In addition to biotic variables, which have been neglected for years in metacommunity studies, the loss of environmental information due to model selection in GAMs, may give rise to misleadingly high proportions of unexplained residual variation (and/or of pure spatial effects) (Livingston et al., 2017).

Despite some potential limitations, we found that environment and space‐related processes (e.g., selection and dispersal) played an important role in structuring metacommunities of tropical and temperate temporary ponds, although environmental effects dominated in the mediterranean metacommunity (i.e., in the more heterogeneous limnological environment). Contrary to expectations underlying spatial processes, the type of dispersal ability of the studied taxa (active or passive) did not relate to the proportion of spatial or environmental control on their metacommunities. We found no general pattern among taxonomic groups between regions, with very variable and idiosyncratic responses, even though mediterranean organisms responded more similarly to those patterns described in the literature; organisms are usually more environmentally than spatially controlled, and more so in temperate than in tropical metacommunities. This is however a snapshot study at a time characterized by high stochasticity at the beginning of the hydroperiod. To further disentangle the actual role of each process in shaping metacommunities, we need to study their temporal dynamics to better quantify not only the relative role of environmental and spatial components along a temporal series, but also the role of time per se in structuring metacommunities in highly dynamic ecosystems (Gálvez et al., 2020; Gomes‐Mello et al., 2021; Rojo et al., 2016).

## CONFLICT OF INTEREST

The authors declare no conflict of interest.

## Supporting information


Appendix S1

## Data Availability

Environmental characterization data and community matrices (Gálvez & Mesquita, 2021) are available in Figshare at http://doi.org/10.6084/m9.figshare.14644608.v4. Codes for variation partitioning analyses (Gálvez et al., 2022) are available in Zenodo at https://doi.org/10.5281/zenodo.6883482.
