# Supplementary material for: Inconsistent response of taxonomic groups to space and environment in mediterranean and tropical pond metacommunities
Source: Ecology. 2022 Oct 5;104(1):e3835. doi: 10.1002/ecy.3835 (PMC10078490; doi:10.1002/ecy.3835)

Ecology

Appendix S1

Inconsistent response of taxonomic groups to space and environment in mediterranean and tropical pond metacommunities

Ángel Gálvez, Pedro R. Peres-Neto, Andreu Castillo-Escrivà, Fabián Bonilla, Antonio Camacho, Eduardo M. García-Roger, Sanda Iepure, Javier Miralles, Juan S. Monrós, Carla Olmo, Antonio Picazo, Carmen Rojo, Juan Rueda, María Sahuquillo, Mahmood Sasa, Mati Segura, Xavier Armengol, Francesc Mesquita-Joanes

Table S1: Results of variation partitioning analysis for each group. The table shows the proportion of variation (R^2_{adj}) explained by each pure component (E|S and S|E), the overlaps between components or spatialized environment ($E \cap S$), and the total explained variation (E+S) in tropical and mediterranean metacommunities.

Group of organisms	E|S		$E \cap S$		S|E		E+S	
Region	Trop	Med	Trop	Med	Trop	Med	Trop	Med
Water Bacteria	0.03	0.42	0.40	0.10	0.09	0.04	0.52	0.56
Sediment Bacteria	0.09	0.29	0.27	0.00	0.26	0.17	0.62	0.46
Water Archaea	0.19	0.48	0.01	0.00	0.36	0.00	0.56	0.48
Sediment Archaea	0.05	0.08	0.26	0.00	0.13	0.00	0.45	0.08
Phytoplankton	0.23	0.35	0.05	0.10	0.14	0.00	0.41	0.45
Cyanobacteria	0.05	0.71	0.06	0.00	0.29	0.00	0.40	0.71
Chlorophyceae	0.33	0.24	0.00	0.00	0.12	0.00	0.45	0.24
Mixotrophic flagellate	0.00	0.09	0.16	0.01	0.01	0.15	0.17	0.26
Bacillariophyceae	0.29	0.19	0.00	0.02	0.00	0.53	0.29	0.74
Rotifera	0.22	0.75	0.13	0.10	0.16	0.00	0.51	0.85
Microcrustaceans	0.10	0.45	0.26	0.02	0.22	0.05	0.58	0.52
Branchiopoda	0.10	0.46	0.16	0.00	0.00	0.00	0.26	0.46
Copepoda	0.13	0.44	0.10	0.00	0.00	0.00	0.23	0.44
Ostracoda	0.18	0.18	0.23	0.00	0.00	0.33	0.42	0.51
Macroinvertebrates	0.13	0.66	0.10	0.00	0.29	0.00	0.52	0.66
Insecta	0.02	0.63	0.07	0.03	0.18	0.14	0.27	0.81
Palaeoptera	0.31	0.28	0.00	0.00	0.00	0.56	0.31	0.85
Heteroptera	0.28	0.00	0.02	0.00	0.05	0.52	0.34	0.52
Coleoptera	0.14	0.13	0.00	0.29	0.00	0.11	0.14	0.53
Diptera	0.07	0.71	0.17	0.00	0.35	0.00	0.59	0.71
Chironomidae	0.24	0.76	0.26	0.00	0.27	0.00	0.77	0.76
Mollusca	0.00	0.10	0.00	0.00	0.10	0.00	0.10	0.10
Amphibia	0.00	0.28	0.61	0.00	0.08	0.00	0.70	0.28
Aves	0.00	0.20	0.41	0.42	0.11	0.31	0.53	0.93

Figure S1: Results of variation partitioning analysis for each group of organisms in tropical and Mediterranean metacommunities. The proportion of variation explained by each component is represented with a different color. Taxa in bold type include species from the following groups enclosed in the corresponding line. Red dashed line represents average total explained variation. Number of identified species (Tropical | Mediterranean species) are shown next to each group label.

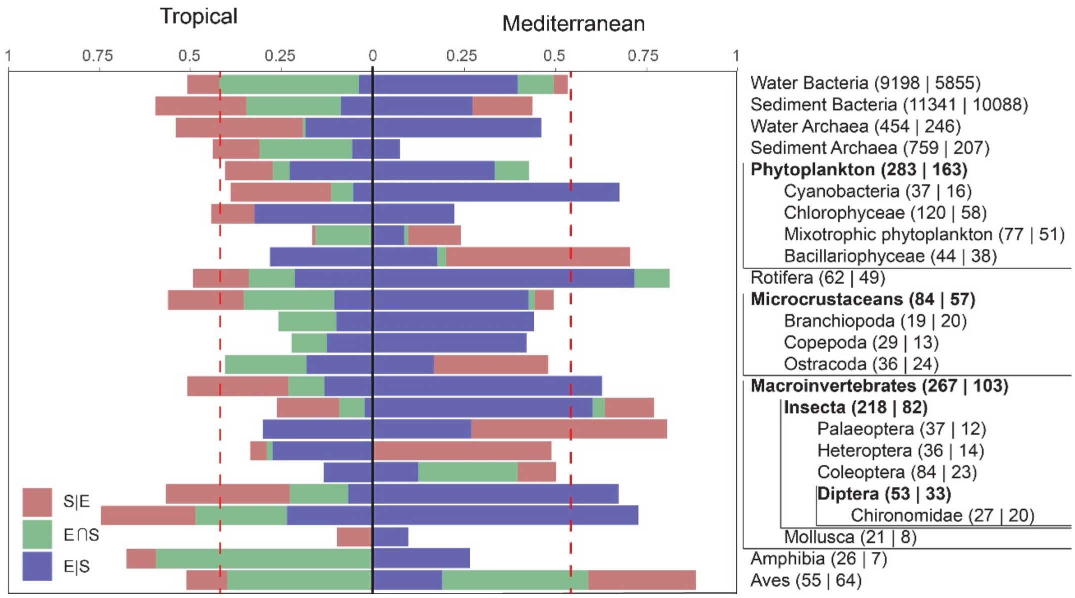

Supplement: Supplementary file 1 — Appendix S1 [file ECY-104-0-s001.pdf]
